# Human Endogenous Retrovirus K(HML-2) Gag and Env specific T-cell responses are not detected in HTLV-I-infected subjects using standard peptide screening methods

**DOI:** 10.1186/1477-5751-12-3

**Published:** 2013-01-10

**Authors:** R Brad Jones, Fabio E Leal, Aaron M Hasenkrug, Aluisio C Segurado, Douglas F Nixon, Mario A Ostrowski, Esper G Kallas

**Affiliations:** 1 Department of Immunology, University of Toronto, 1 King's College Circle, Rm 6352, Toronto, ON, M5S 1A8, Canada; 2 Ka Shing Knowledge Institute of St. Michael’s Hospital, Toronto, ON, Canada; 3 Division of Clinical Immunology and Allergy, Department of Infectious Diseases, School of Medicine, University of Sao Paulo, Sao Paulo, Brazil; 4 Department of Infectious Diseases, School of Medicine, University of Sao Paulo, Sao Paulo, Brazil; 5 Division of Experimental Medicine, University of California San Francisco, San Francisco, CA, USA

**Keywords:** HTLV-I, Human endogenous retrovirus, T-cells, HTLV-1-associated myelopathy/tropical spastic paraparesis

## Abstract

**Background:**

An estimated 10–20 million individuals are infected with the retrovirus human T-cell leukemia virus type 1 (HTLV-1). While the majority of these individuals remain asymptomatic, 0.3-4% develop a neurodegenerative inflammatory disease, termed HTLV-1-associated myelopathy/tropical spastic paraparesis (HAM/TSP). HAM/TSP results in the progressive demyelination of the central nervous system and is a differential diagnosis of multiple sclerosis (MS). The etiology of HAM/TSP is unclear, but evidence points to a role for CNS-inflitrating T-cells in pathogenesis. Recently, the HTLV-1-Tax protein has been shown to induce transcription of the human endogenous retrovirus (HERV) families W, H and K. Intriguingly, numerous studies have implicated these same HERV families in MS, though this association remains controversial.

**Results:**

Here, we explore the hypothesis that HTLV-1-infection results in the induction of HERV antigen expression and the elicitation of HERV-specific T-cells responses which, in turn, may be reactive against neurons and other tissues. PBMC from 15 HTLV-1-infected subjects, 5 of whom presented with HAM/TSP, were comprehensively screened for T-cell responses to overlapping peptides spanning HERV-K(HML-2) Gag and Env. In addition, we screened for responses to peptides derived from diverse HERV families, selected based on predicted binding to predicted optimal epitopes. We observed a lack of responses to each of these peptide sets.

**Conclusions:**

Thus, although the limited scope of our screening prevents us from conclusively disproving our hypothesis, the current study does not provide data supporting a role for HERV-specific T-cell responses in HTLV-1 associated immunopathology.

## Background

Human T-cell leukemia virus type 1 (HTLV-1) is a deltaretrovirus that has infected humans for thousands of years. An estimated 10–20 million individuals worldwide are infected with HTLV-1, the majority of whom will remain asymptomatic for life [1]. For a minority of infected individuals, however, HTLV-1 infection is associated with severe diseases, with 1-5% developing adult T-cell leukemia/lymphoma [2] (ALT) and an additional 0.3-4% developing immune-mediated inflammatory disease involving the central nervous system, the eyes, the lungs and/or the skeletal muscles [3]. Inflammation of the central nervous system (CNS) in HTLV-1-infected subjects results in progressive spasticity and muscle weakness in lower extremities and is termed HTLV-1-associated myelopathy/tropical spastic paraparesis (HAM/TSP) [4]. The etiology of HAM/TSP is poorly understood, however it is associated with higher HTLV-1 proviral loads, a higher frequency of HTLV-1-specific CD8^+^ T-cells and greater production of IFN-γ and TNF-α than that observed in asymptomatic HTLV-1-infected subjects, suggesting the involvement of cellular immune responses in the pathogenesis [5-8]. As there is no evidence that HTLV-1 infects astrocytes, microglia or neuronal cells, it is unlikely that the damage incurred by these cell populations is mediated by direct recognition of HTLV-1 antigens presented on these cells by HTLV-1-specific CD8^+^ T-cells. Two potential mechanisms have been proposed to explain how HTLV-1-specific CD8^+^ T-cells could mediate damage to non-HTLV-1-infected cells. Firstly, this damage could occur simply as a bystander effect of HTLV-1-specific CD8^+^ T-cells recognizing the HTLV-1-infected T-cells that have been shown to accumulate in the CNS. Secondly, it has been suggested that there may be cross-reactivity of HTLV-1-specific CD8^+^ T-cells with self antigens expressed in neurons [9]. Here, we present and explore a third possibility, that HTLV-1-infection may elicit human endogenous retrovirus (HERV) specific CD8^+^ T-cell responses which, in turn, may be reactive against neurons and other tissues where HERV antigens may be expressed.

Human endogenous retroviruses, the DNA remnants of ancient infectious retroviruses, comprise approximately 8% of the human genome. This complement of HERVs is diverse, with all three major branches of the retroviral tree represented: gamma-epsilon, spuma and delta-lenti-alpha-beta retroviruses [10]. While the vast majority of HERV insertions do not contain intact open reading frames (ORFs), a recent study has illustrated the potential for even short, disrupted ORFs to serve as a source of immunologically relevant antigens, by mapping the epitope specificity of a renal cell carcinoma reactive CD8^+^ T-cell to a HERV-E-derived peptide [11]. As a gross generalization, HERV antigens are not thought to be expressed in healthy tissues, but rather are associated with transformed cell lines and disease states. We have previously presented the hypothesis that HIV-1 infection would result in the induction of HERV expression, either through rescue of HERV function by HIV-1, for example, nuclear export of unspliced HERV RNA by HIV-1 Rev, or through the degradation of host restriction factors which may normally keep HERV expression in check [12]. Indirect support for this hypothesis is provided by our observation that T-cell responses to peptides derived from diverse HERV families can be detected in HIV-1-infected, but not uninfected, subjects [12-14]. We have since focused in on the HERV-K(HML-2) lineage of young and relatively intact endogenous retroviruses, and determined that HIV-1 infection results in the induction of Gag and Env protein expression. A HERV-K(HML-2)-Env-specific CD8^+^ T-cell clone, isolated from an HIV-1-infected subject, specifically responded to and eliminated cells infected with diverse isolates of HIV-1, HIV-2 and SIV [15]. The establishment of this precedent for induction of HERV expression by an exogenous retroviruses led us to consider whether HTLV-1, as a second human retrovirus, may also drive the expression of HERVs. This idea draws support from a very recent study which demonstrated that the HTLV-1-Tax protein drives transcription from the long-terminal repeats (LTR) of HERV-W8, HERV-H and HERV-K [16]. In the current study we sought to obtain indirect evidence for the *in vivo* expression of HERV antigens, by screening PBMC from HTLV-1-infected subjects for T-cell responses to HERV-derived peptides, with a particular focus on the HERV-K(HML-2) lineage.

In addition to providing evidence for the HTLV- 1-induced expression of HERVs, the detection of HERV-specific T-cell responses in HTLV-1-infected subjects would open a line of inquiry into determining whether such responses may contribute to the pathogenesis of HAM/TSP. It is intriguing to note, given the similarities in clinical presentation and pathogenesis of HAM/TSP and multiple sclerosis (MS), that numerous studies have associated HERV polymorphisms or expression levels with MS (reviewed in [17]). Specifically, HERV-H, HERV-W, and HERV-K have been associated with MS, the same families shown to be induced at the transcriptional level by HTLV- 1-Tax. We posited that HTLV-1 infection would result in the expression of HERV antigens and the induction of HERV-specific T-cell responses, which would, in turn, be reactive against neurons and other tissues which may express low levels of HERV antigens, resulting in the pathologies of HAM/TSP. Consistent with this, we have recently performed a comprehensive screening of human tissues for HERV-K(HML-2)-Gag expression and detected this protein in the central nervous system (neurons, purkinje cells and occasionally ependymal cells), as well as in the endocrine pancreas (few cells in the β-islets of Langerhans) and in the exocrine pancreas (ductal epithelial cells) (Sacha *et al*. Submitted).

## Results and discussion

To test this hypothesis, we obtained peripheral blood mononuclear cells (PBMC) from 15 HTLV-1-infected subjects from Sao Paulo, Brazil, and from 8 age/sex matched uninfected subjects from the same geographical area. The HTLV-1-infected subjects comprised 9 individuals who were asymptomatic, 5 with HAM/TSP and 1 subject who we define as pre-ATL. In the latter subject, significant immunophenotype alterations were present at the time of sample collection, but the criteria for an ATL diagnosis were not present. Six months later, this patient was diagnosed with the lymphomatous variant of ATL (Table 1). These PBMC were screened for T-cell responses by IFN-γ ELISPOT. The primary objective of these experiments was to comprehensively screen for responses to HERV-K(HML-2) Gag and Env using overlapping peptide pools spanning these antigens. We did also screen these subjects for responses to peptides derived from diverse HERV families selected based on predicted binding to common MHC-I alleles, many of which have previously been demonstrated to be immunoreactive HIV-1-infected subjects [12-14]. However, as we had no prior knowledge of the HLA-types of subjects in our cohort, the latter screening was performed without consideration of whether subjects possessed the necessary restricting alleles, imposing limitations on our ability to draw conclusions from these data. Peptides tested were thus: i) 15mer peptides, overlapping by 11 amino acids, spanning HERV-K(HML-2) Gag and Env – tested in pools of 10 peptides each at 25 μg/ml/peptide ii) 29 shorter peptides (9–13 amino acids) derived from HERV-K(HML-2), HERV-L, HERV-H and HERV-W (Table 2) – tested in pools of 7 peptides each at 10 μg/ml/peptide. These peptides have been previously described [12-14], and were selected based on predicted binding to the MHC-I alleles A02, B58, B07 or B35 iii) the immunodominant HLA-A02-restricted Tax 11–19 peptide tested at 10 μg/ml iv) a pool of CMV, EBV and influenza derived peptides (CEF pool) as a positive control tested at 2.5 μg/ml/peptide v) *Staphylococcus* enterotoxin B (SEB) tested at 2.5 μg/ml. ELISPOTS were performed as previously described [13], except that stimulation periods were limited to 6 hours. Longer periods of *in vitro* culture of PBMC derived from HTLV-1-infected subjects resulted in very high levels of spontaneous IFN-γ production, as has been previously reported [18].

**Table 1:** Clinical data

**Healthy donors**	**Gender**	**Age**	**Proviral load copies/ 1000 cells**
HD001	F	39	NA
HD002	F	30	NA
HD003	M	21	NA
HD004	M	31	NA
HD005	F	60	NA
HD006	M	51	NA
HD007	M	38	NA
HD008	F	45	NA
**Asymptomatic**			
401	F	56	<1
403	F	55	ND
405	F	23	15
409	M	20	1
416	M	49	1162
419	F	34	72
424	M	46	106
425	M	29	43
426	F	51	101
**HAM/TSP**			
404	M	45	4
407	M	61	357
412	F	55	18
415	F	27	444
420	M	64	12
**Pre ATL**			
413	F	62	3650

**Table 2:** HERV-Opt peptides – selected on basis of predicted binding to common MHC-I alleles

**Antigen**	**Sequence**
HERV-K-Pol	KLIDCYTFL
HERV-K-Gag	KLFQIIEQF
HERV-K-Gag	YPQPPTRRL
HERV-K-Pol	FAFTIPAI
HERV-K-Pol	FEGLVDTGAD
HERV-K-Pol	VPLTKEQVR
HERV-K-Pol	IPTGVYPGL
HERV-L	LQDIILVHY
HERV-L	VVIQITSPFNSPKW
HERV-L	SQGYINSPAL
HERV-L	QINTSPGTW
HERV-L	KAHKKQFAFSW
HERV-L	RSWRMTVDYCKL
HERV-L	IPVHKAHKKQ
HERV-K-Gag	QNIIPLTVW
HERV-H	LDLLTAEKGGLCI
HERV-K-Gag	FLQFKTWWI
HERV-K-Pol	GIPYNSQGQ
HERV-K-Gag	KSSLSPSQF
HERV-K-Pol	VSIIALNQW
HERV-K-Gag	RLIPYDWEI
HERV-W	DSIEGQLILK
HERV-C	TLEPIPPGE
HERV-L	KIRLPPGYF
HERV-L	ILVHYIDDI
HERV-L	AAIDLANAF
HERV-L	PMVSTPATL
HERV-L	SSGLMLMEF

We observed a total of 12 HERV-specific responses in HTLV-1-infected individuals which passed our pre-established criteria of > 50 sfu/10^6^ PBMC after background subtraction and > 3x background versus 3 HERV-specific responses in uninfected subjects. These responses were, however, of borderline magnitude and replicates between duplicate wells were poor. We sought to repeat these responses with new aliquots of cells from the same patient time-points using the same peptide pools and, in parallel, to deconvolute responses to pools by testing individual peptides. In these follow-up experiments we observed that none of these responses were reproducible. The incidence of these false positive responses was related to ‘noisier’ ELISPOTs than those typically observed using PBMC from HTLV-1-uninfected subjects. Although we sought to minimize the impact of spontaneous IFN-γ production on the assay by reducing stimulation periods to 6 hours, we observed higher levels of background in ELISPOTs performed on HTLV- 1-infected subjects than in uninfected controls. Despite this background, we observed clear and reproducible responses to the HLA-A02-restricted Tax peptide in 6 HTLV-1 subjects, and an absence of this response in uninfected subjects. Responses to CEF pool were detected in the majority of both HTLV-1-infected and uninfected subjects.

**Figure 1:**
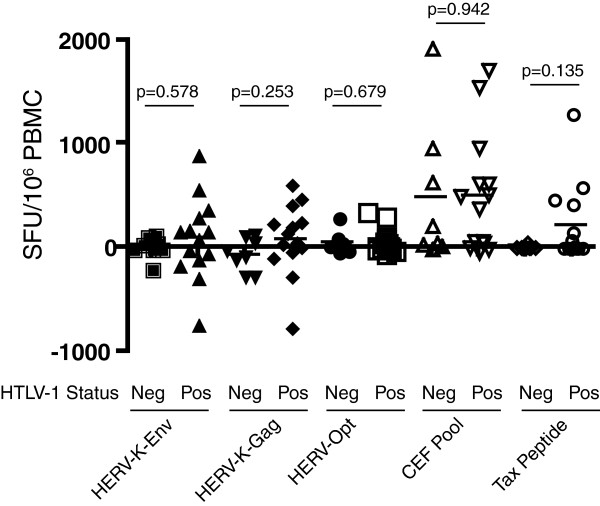
**Summary ELISPOT data. **PBMC from HTLV-1-infected and uninfected subjects were screened with the indicated peptides in IFN-γ ELISPOT assays. In the cases of HERV-K-Env, HERV-K-Gag and HERV-Opt the mean background (0.5% DMSO) subtracted responses to individual peptide pools (18 pools for Env, 17 pools for Gag, 4 pools for Opt) were summed to give a total response. Responses are expressed as spot forming units (SFU) per million PBMC. P values were calculated using Student’s T-test.

We next summed the background-subtracted responses to: HERV-K(HML-2)-Env, HERV-K(HML-2)-Gag and predicted optimal HERV peptides (HERV-Opt). In screens performed on non-HTLV-1-infected subjects ELISPOT results with negative values after background subtraction are atypical and generally reported as zero. In the present case, however, the variability caused by spontaneous cytokine production, and summed over multiple peptide pools in the cases of HERV-K(HML-2)-Gag and Env (17 and 18 pools respectively), manifested as cumulative totals which spanned a range from substantially more than to less than zero. As zeroing these negative values would give the false impression of positive responses in HTLV-1-infected subjects (instead of just cumulative noise) these data are presented as is. We observed a lack of statistically significant differences between the magnitudes of responses detected in HTLV-1-infected versus uninfected subjects for each of the peptide sets tested (Figure 1). Notably, however, we did observe a trend towards the detection of responses to the HLA-A02-restricted Tax peptide in HTLV-1-infected subjects (p=0.135). The failure of this response to reach significance is likely tied to the fact that subjects were tested without a prior knowledge of their HLA types. Thus, only a minority (approximately 40%) of subjects would be expected to have the capacity to present this particular peptide. A lack of statistically significant differences in magnitudes of responses to HERV, Tax or CEF peptides were also observed when we broke the HTLV-1-infected subjects into asymptomatic versus HAM/TSP groups (data not shown). We did, however, observe a positive correlation between the magnitude of responses to the HLA-A02-restricted Tax peptide and HTLV-1 proviral load (considering all subjects, not just responders) (R=0.785, p=0.002, Table 3). This is in agreement with observations from two previous reports [19,20]. We did not observe any such correlation with magnitudes of responses to HERV-K(HML-2) Env or Gag, to the optimized HERV peptide pool or to CEF (Table 3). Thus the data are indicative of a lack of detectable T-cell responses to the HERV-derived peptides considered in the present study.

**Table 3:** Correlations between magnitudes of responses and HTLV-I proviral loads

**Peptides**	**R value**	**P value**
HERV-K-Env	0.141	0.647
HERV-K-Gag	0.199	0.515
HERV-Opt	0.098	0.751
CEF Pool	0.023	0.940
Tax Peptide	0.785	0.002
SEB	0.158	0.606

## Conclusions

The current study does not provide any data to support the hypothesis that the induction of HERV expression and HERV-specific T-cell responses in HTLV-1-infected subjects may play a role in the pathogenesis of HAM/TSP. This may indicate that, in contrast to HIV-1, HTLV-1 does not induce HERV expression. We have recently demonstrated that the HIV-1-Vif protein is necessary for the induction of HERV-K(HML-2) expression [15], thus the lack of a Vif equivalent in HTLV-1 may at least partially underlie this difference. There are a number of important caveats, however, which prevent the current study from comprehensively ruling out a role for HERVs in HTLV-1 pathogenesis. Firstly, the HERV-derived peptides tested in the current study only represent a small fraction of the total HERV antigens encoded in the human genome. In particular, HERV-H and HERV-W, which were shown to be transcriptionally induced to the greatest level by Tax in a recent study [16], were only represented by one peptide each (this study was published after our experiments were completed). Secondly, the HERV peptides, predicted to be optimal binders for either HLA-A02, B07, B58 or B35, were tested without a prior knowledge of the HLA-types of the test subjects in this cohort. HLA-A02 is common enough (~40% prevalence in this cohort) that responses to immunodominant HLA-A02-restricted HERV-derived peptides would likely have been detected in this cohort, as is evidenced by our detection of responses to the HLA-A02-restricted Tax peptide. However, the detection of responses restricted by the less common B07, B58, or B35 alleles was unlikely. Indeed, these were tested as an adjunct to the primary objective of the current study, which was to comprehensively screen for responses to HERV-K(HML-2) Gag and Env. Importantly, in our previous studies measuring responses to these predicted optimal epitopes in HIV-1-infected subjects, these peptides were tested against a cohort of subjects selected on the basis of having one or more of these restricting alleles. In the current manuscript, we do comprehensively screen for T-cell responses to HERV-K(HML-2) Gag and Env using overlapping 15mer peptides. This approach is not reliant upon prior knowledge of a subject’s HLA-type, and yet a lack of T-cell responses was still observed. When we have previously screened HIV-1-infected subjects with these same peptides we observed only infrequent responses, with 3 responses detected in the 27 subjects who were screened [21]. Thirdly, the peptides used in the current study represent only a small fraction of the potential HERV antigens encoded by 8% of our genome. The current study is best interpreted as a starting point, effectively ruling out the presence of responses to HERV-K(HML-2) Gag and Env that are detectable using 15mer peptide screens. The recent demonstration that HERV-H and HERV-W are most potently induced at the transcriptional level by HTLV-1-Tax, with a lesser induction of HERV-K, suggests that future studies should focus on screening for T-cell responses to these HERV families. Finally, while we have focused exclusively on T-cell responses as potential mediators, there are also other potential mechanisms by which putative HTLV-1-mediated HERV induction could result in pathogenesis – for example, through antibody mediated immunopathology or through direct effects of HERV gene products on the affected tissue (toxicities of Env proteins for example). Although limited by the caveats given above, the current study argues against a role for an involvement of HERVs in the immunopathogenesis of HTLV-I infection.

## Methods

Samples were collected under an IRB approved protocol. PBMC were isolated through Ficoll gradient enrichment and cryopreserved. ELISPOT assays were performed as previously described [21], but with a 6 hour, rather than overnight, stimulation period. Peptides were tested as follows: 15mer peptides, overlapping by 11 amino acids, spanning HERV-K(HML-2) Gag and Env – tested in pools of 10 peptides each at 25 μg/ml/peptide ii) 29 shorter peptides (9–13 amino acids) derived from HERV-K(HML-2), HERV-L, HERV-H and HERV-W (Table 2) – tested in pools of 7 peptides each at 10 μg/ml/peptide iii) the immunodominant HLA-A02-restricted Tax 11–19 peptide tested at 10 μg/ml iv) a pool of CMV, EBV and influenza derived peptides (CEF pool) tested at 2.5 μg/ml/peptide. *Staphylococcus* enterotoxin B (SEB) was used at 2.5 μg/ml. A response was considered to be positive if it passed both the criteria of: i) > 50 sfu/million PBMC after background subtraction and ii) > 3x background. Statistics were performed using Prism Graphpad 2.0 software.

## Abbreviations

HTLV-1: Human T-cell leukemia virus type 1; HERV: Human endogenous retrovirus; HAM/TSP: HTLV-1-associated myelopathy/tropical spastic paraparesis; MS: Multiple sclerosis; HIV-1: Human immunodeficiency virus type 1; PBMC: Peripheral blood mononuclear cells; CEF: Cytomegalovirus, epstein barr virus, influenza (peptide pool); ATL: Adult T-cell leukemia; HLA: Human leukocyte antigen.

## Competing interests

The authors declare that they have no competing financial interests.

## Authors’ contributions

RBJ, FEL, DFN, MAO, EGK conceived of the project and designed experiments. FEL, ACS recruited study participants and AMH processed samples. RBJ, AMH, MAO performed the ELISPOT assays. RBJ and AMH analyzed the ELISPOT data. DFN, MAO, EGK supervised the project. Experiments were performed in the laboratory of EGK. RBJ wrote the manuscript with input from all co-authors. All authors read and approved the final manuscript.
